# Maternal Serologic Screening to Prevent Congenital Toxoplasmosis: A Decision-Analytic Economic Model

**DOI:** 10.1371/journal.pntd.0001333

**Published:** 2011-09-27

**Authors:** Eileen Stillwaggon, Christopher S. Carrier, Mari Sautter, Rima McLeod

**Affiliations:** 1 Department of Economics, Gettysburg College, Gettysburg, Pennsylvania, United States of America; 2 Division of Ophthalmology and Visual Sciences, Department of Surgery, The University of Chicago, Chicago, Illinois, United States of America; Imperial College London, United Kingdom

## Abstract

**Objective:**

To determine a cost-minimizing option for congenital toxoplasmosis in the United States.

**Methodology/Principal Findings:**

A decision-analytic and cost-minimization model was constructed to compare monthly maternal serological screening, prenatal treatment, and post-natal follow-up and treatment according to the current French (Paris) protocol, versus no systematic screening or perinatal treatment. Costs are based on published estimates of lifetime societal costs of developmental disabilities and current diagnostic and treatment costs. Probabilities are based on published results and clinical practice in the United States and France. One- and two-way sensitivity analyses are used to evaluate robustness of results. Universal monthly maternal screening for congenital toxoplasmosis with follow-up and treatment, following the French protocol, is found to be cost-saving, with savings of $620 per child screened. Results are robust to changes in test costs, value of statistical life, seroprevalence in women of childbearing age, fetal loss due to amniocentesis, and to bivariate analysis of test costs and incidence of primary *T. gondii* infection in pregnancy. Given the parameters in this model and a maternal screening test cost of $12, screening is cost-saving for rates of congenital infection above 1 per 10,000 live births. If universal testing generates economies of scale in diagnostic tools—lowering test costs to about $2 per test—universal screening is cost-saving at rates of congenital infection well below the lowest reported rates in the United States of 1 per 10,000 live births.

**Conclusion/Significance:**

Universal screening according to the French protocol is cost saving for the US population within broad parameters for costs and probabilities.

## Author Summary

We constructed a decision-analytic and cost-minimization model to compare monthly maternal serological screening for congenital toxoplasmosis, prenatal treatment, and post-natal follow-up and treatment according to the current French protocol, versus no systematic screening or perinatal treatment. Costs are based on published estimates of lifetime societal costs of developmental disabilities and current diagnostic and treatment costs. Probabilities are based on published results and clinical practice in the United States and France. We use sensitivity analysis to evaluate robustness of results. We find that universal monthly maternal screening for congenital toxoplasmosis with follow-up and treatment, following the French (Paris) protocol, leads to savings of $620 per child screened. Results are robust to changes in test costs, value of statistical life, seroprevalence in women of childbearing age, fetal loss due to amniocentesis, incidence of primary *T. gondii* infection during pregnancy, and to bivariate analysis of test costs and incidence of primary *T. gondii* infection. Given the parameters in this model and a maternal screening test cost of $12, screening is cost-saving for rates of congenital infection above 1 per 10,000 live births. Universal screening according to the French protocol is cost saving for the US population within broad parameters for costs and probabilities.

## Introduction

*Toxoplasma gondii* infects between one third and one half of the world's population, usually without recognized symptoms. Congenital toxoplasmosis (CT), however, transmitted transplacentally from mother to fetus, can have serious and potentially devastating effects in virtually all infected, untreated children at varying times in their lives. CT is not a reportable disease in the United States, nor is a maternal screening program for toxoplasmosis routinely offered by most health care providers. It is estimated that between 400 and 4,000 infected children are born each year in the United States, some of whom suffer severe recurring and progressive visual symptoms, as well as hearing, motor, and cognitive impairments, and seizures [1]–[12].

In France, a nationally mandated detection and treatment program has reduced rates and severity of congenital infections, such that severe CT, as seen in the United States, is only rarely encountered [9], [13]–[22]. The French program requires monthly serologic screening for congenital toxoplasmosis usually beginning by the 11^th^ week of gestation for the duration of pregnancy for all seronegative at-risk mothers. The question addressed herein is whether and, if so, under what circumstances, screening for congenital toxoplasmosis according to the French protocol would be a cost-saving intervention in the United States. Through the development of a decision-analytic model, cost estimates for a “screening” strategy and “no screening” strategy are generated and compared.

Congenital toxoplasmosis is a neglected infection in the United States, particularly for women with inadequate prenatal care, and can be a devastating disease in developing, tropical countries [23], [24]. In the United States, incidence is higher in southern areas with temperate and subtropical climates [25]–[27]. Oocysts can persist for up to a year in warm, moist soil, especially in humid climates. The disease is present in persons of all socioeconomic groups throughout the United States. Some populations, such as Pennsylvania Amish, have particularly high seroprevalence in women of child-bearing age [28], [29]. In developing and developed countries, this infection can be life-threatening and cause prematurity, destruction of the eye, damage to the brain, hydrocephalus, microcephalus, and seizures. For example, in Belo Horizonte, Brazil, one in every 300 babies born has active eye disease due to *T. gondii* [23].

In Europe and in the United States three predominant clonal lineages of *T. gondii* have been described [30], [31]. Type II *T. gondii* is reported to predominate in France, Poland, and the United States [31]. In a small series, atypical genetic types of *T. gondii* are reported to cause unusually severe eye disease in the United States [32]. The presence of types I, III, and atypical *T. gondii* parasites in South and Central America has been associated with significant human disease, and very recently distinct atypical, now called Type IV haplotype, parasites have been found in domestic and wild animals in the United States in substantial numbers [33]–[40]. In certain areas of Brazil, Colombia, and Guatemala, *T. gondii* strains are atypical rather than the three clonal lineages in Europe and the United States and are often genetically polymorphic [41], [42]. Migration of people from Central and South America and travel to and from the United States, with some pregnant women traveling during gestation, may also contribute to disease in the United States. The presence of environmental contamination by oocysts is a common source of infection in temperate climates, and the migratory patterns of birds that feed on ground where oocysts are present might account for some of the atypical genetic strains of *T.gondii* that have been noted in the United States [43]. Furthermore, the globalization of markets with exchange of food products between North and South America may result in consumption of meat, fruits, and vegetables contaminated with oocysts from types I, III and atypical parasites. It is also postulated that global warming may lead to an increase in incidence of the infection in some areas of the world [44].

### Background Data Concerning Epidemiology, Pathology, Transmission, and Clinical Manifestations of Congenital Toxoplasmosis

Toxoplasmosis is a disease caused by the protozoan parasite *Toxoplasma gondii,* whose definitive host is the cat. Prevalence of toxoplasmosis varies greatly across geographic regions, specifically in relation to differences in climate, dietary practices, and hygiene. The most recent reliable estimate of *T. gondii* seroprevalence in the United States is derived from the National Health and Nutrition Examination Survey (NHANES 1999–2004). In that survey, sera were obtained from a cluster sample of US residents and tested by the Centers for Disease Control and Prevention (CDC) for *T. gondii* antibodies. Of nearly 16,000 persons tested, aged 6–49 years old, age-adjusted seroprevalence was 10.8%, and among women aged 15–44 years, it was 11% [45]. Thus, 89% of US women remain susceptible to acute *Toxoplasma* infection during childbearing years, and their children are at risk for congenital toxoplasmosis.

Estimates of incidence of congenital toxoplasmosis are derived primarily from three studies conducted in the United States. In the 1970s, two prospective studies reported rates of congenital infection to be 10 in 10,000 live births [46], [47]. Data from two surveys in Chicago in the 1980s suggest that incidence of CT was 9 in 10,000 live births [48]. More recently, data from the New England Regional Newborn Screening Program suggest that congenital infection was detected in 1 in 10,000 live births [49], [50], using a test similar to one that identifies 50% of infections [51], [52]. Extrapolated to the approximately 4 million births in the United States each year, an estimated 400–4,000 infants are born each year with congenital toxoplasmosis [25].

In the United States, meat (particularly pork and lamb) has been identified as an important source of infection, yet the proportion of infection derived from meat versus gardening, eating raw or unwashed vegetables, exposure to cat feces, poor hand hygiene, and other routes that go unrecognized is not known [25]. Epidemiological studies of an outbreak of toxoplasmosis in western Canada in the 1990s implicated the municipal water supply as the source of infection [53]. Other water-borne outbreaks also have been reported [54],[55].

Most often, congenital transmission occurs in mothers who acquire primary infection during gestation, although in rare cases congenital transmission has occurred due to the reactivation of a chronic infection in women who are immunocompromised (due to AIDS or various medical treatments) with subsequent congenital transmission [56], [57]. Clinical evidence suggests that *T. gondii* may be present in the placenta for a number of weeks before being transmitted to the fetus, with an observed range from 4 to 16 weeks [57]. The majority of mothers who acquire acute infection during pregnancy fail to display any obvious symptoms, although a minority may present with malaise, low-grade fever, or lymphadenopathy [5], [56].

Not all mothers who become infected with *T. gondii* transmit the infection to the fetus; frequency of vertical transmission increases with gestational age. Risk of transmission of maternal infection acquired before conception is virtually zero and transmission rates remain low for approximately the first 10 weeks. After that, the rate of transmission increases sharply, resulting in a steeply increasing incidence of congenital infection, with 2/3 of mothers transmitting after 30 weeks gestation [57].

While infected pregnant women typically present no symptoms, congenital infection may cause fetal death or injuries including vision and hearing deficits, cognitive impairment, or central nervous system lesions. Congenital *T. gondii* infections have varied manifestations, including symptomatic neonatal disease, with prematurity, rash, thrombocytopenia, illness mimicking out sepsis (rule out sepsis), jaundice, hepatosplenomegaly, hepatitis, anemia, leukopenia or leukocytosis, seizures, meningitis, encephalitis, chorioretinitis or chorioretinal scars, vision loss, intracranial calcifications, hydrocephalus, and microcephalus. Disease, from mild to severe, may manifest within one month of birth or not until childhood or adolescent sequelae from previously undiagnosed infections become apparent, or may include subclinical infection [5], [6], [13].

The risk of severe disease is greater when maternal infection is acquired in the first or second trimester [13]. Despite higher rates of transmission of maternal infection to the fetus in the third trimester, transmission that occurs later in pregnancy generally results in subclinical infection or milder manifestations of congenital toxoplasmosis at birth.

### Prevention Options

Congenital toxoplasmosis can be prevented only by preventing maternal infection or by stopping transmission from mother to fetus. Preconceptional and early pregnancy counseling can help women avoid personal exposure to *T. gondii* in undercooked food or material potentially contaminated by cat excrement. A 1994 study of toxoplasmosis in Belgium found that preconceptional education was associated with a 63% reduction in the rate of seroconversion [58]. Other studies, however, have found that mothers giving birth to congenitally infected infants in the United States commonly do not recognize risk factors for which education would have been effective [59].

Blocking transmission from mother to fetus by treating mothers with acute infection is a second way to prevent fetal infection. Maternal treatment is effective in blocking transmission in up to 60% of treated mothers [22], [60]. If transplacental transmission occurs, manifestations of fetal infection can be managed and reduced substantially by diagnosing and treating fetal infection *in utero*.

Early diagnosis and treatment of neonates and older children to treat manifest disease or to attempt to prevent disease progression is another option. With universal neonatal screening, intervention to treat neonates and children who present with symptoms has been found to be cost saving [61].

Some proponents of screening programs advocate universal screening of neonates, whereas others emphasize treating only infants who present with symptoms of acute infection, or even not treating at all in the absence of data from placebo-controlled randomized clinical trials that demonstrate efficacy [9], [14], [20], [57], [62], [63]. In most cases, congenital infection is subclinical at birth, although sequelae develop over time and may cause damage later in life. Neonatal screening can be achieved at relatively low cost by expanding established newborn screening programs to include tests for toxoplasmosis [49], [64]. The clear disadvantage of neonatal screening is that it cannot prevent injury sustained before birth, which may be permanent and profound.

#### Universal Prenatal Screening

In some countries, including Austria, France, and Belgium, systematic prenatal screening for toxoplasmosis is mandated by law to facilitate early detection of recently acquired infection in pregnant women. Screening provides medical benefits of early treatment [6], [9], but arguments against screening include factors such as cost, demographic characteristics, availability of appropriate tests, and the low incidence of acute infection.

Clinical or epidemiological grounds are not sufficient to establish when serologic testing is needed because infection often occurs in asymptomatic pregnant women with no knowledge of direct exposure [59]. Maternal serological testing relies on the detection and quantification of *T. gondii* antibodies to determine whether a pregnant woman is infected with toxoplasmosis and whether the infection was acquired recently. If testing indicates that infection occurred during gestation or shortly before conception, the fetus is at risk. Initial screening at clinical laboratories involves serological testing for IgG (immunoglobulin G) and, if IgG is positive, for IgM (immunoglobulin M) antibodies. Women who test negative for *T. gondii* infection continue to be at risk of acquiring acute infection and are tested monthly. In the event of a positive test result before the 11^th^ week of gestation, reference laboratories are able to confirm a positive IgM test result, and using the avidity method and *Toxoplasma* serological profile (TSP) to determine whether infection occurred before or after conception, and thus decrease the need for follow-up samples and unnecessary additional testing and/or treatment [56]. After 18 weeks, fetal infection can be confirmed through the use of PCR (polymerase chain reaction) of amniotic fluid [56] with greater than 92% sensitivity and 100% specificity [65].

#### Treatment

Treatment of congenital toxoplasmosis has been shown to reduce severity of symptoms of active infection and to improve outcomes [5], [6], [12]. Early diagnosis and treatment minimizes the time for tissue destruction by the parasite. Accordingly, favorable outcomes are associated with treating congenital infection during gestation and infancy [9]. A prospective study in 14 European centers found that prenatal treatment reduced the risk of serious neurological sequelae of CT with an odds ratio for prenatal treatment of 0.24, adjusted for gestational age at maternal seroconversion [19], [21]. There is significant reduction in sight-threatening retinal disease associated with more rapid introduction of treatment [16], [17].

In France, upon confirmation of acute maternal infection early in gestation, treatment with spiramycin is initiated immediately. Spiramycin does not cross the placenta to the fetus, but is present in high concentrations in the placenta [60], and thus it has been reported to decrease the frequency of vertical transmission, with estimates of the reduction of congenital infection of as much as 60% [22], [60]. Studies suggest that spiramycin is most efficacious if administered shortly after maternal seroconversion, and it remains the recommended treatment option for maternal infection acquired before 18 weeks of gestation.

Treatment with pyrimethamine, sulfadiazine, and folinic acid (PSF) is recommended for patients for whom fetal infection has been confirmed, including when infection is acquired after 18 weeks of gestation, due to the high rates of vertical transmission in the second and third trimesters [56]. PSF is used to treat infection in the fetus directly, and studies indicate that treatment with PSF may markedly reduce disease severity at birth and lifetime sequelae [14], [16]–[18], [20], [21], [56], [66].

#### Systematic Screening in France

A universal monthly screening program for congenital toxoplasmosis mandated in France since 1992 provides the template upon which the model herein is based. In the 1960s, seroprevalence of *T. gondii* was found to be 84% in France. Although seroprevalence decreased to 54% in 1995 and further to 44% in 2004, rates of *T. gondii* infection in France are relatively high compared to the United States and northern Europe [67]. The reasons for the decrease in seroprevalence in France are not fully documented but may include better maternal education for prevention and increased awareness of the risks of undercooked food in the population as a whole [68]. A 1984 study found the prevalence of congenital toxoplasmosis in France to be between 1.9 and 3.2 cases per 1,000 live births [69]. Treatment during gestation to prevent congenital toxoplasmosis in France involved administration of spiramycin and PSF (in the manner described earlier). The introduction of treatment with spiramycin was coincident with an observed 50% reduction in the incidence of fetal infection in each trimester from decades prior to initiation of the treatment protocol [14], [15], [17], [22], [66], [70], [71]. Furthermore, due to the delay in transplacental transmission caused by spiramycin treatment, disease severity at birth was lessened for infection acquired in each trimester [72]. Treatment with PSF was similarly effective at reducing disease severity at birth and subsequent sequelae [14], [66].

After 15 years of the universal screening program, the results of a national laboratory surveillance system found the overall prevalence of congenital toxoplasmosis in France in 2007 to be 2.9 to 3.2 per 10,000 live births, and incidence of symptomatic congenital infection of 0.34 per 10,000 live births [67]. There has been an 87% decline in the rate of congenital infection in France since the 1970s, although it is difficult to distinguish between the effect of the reduction in seroprevalence by nearly 50% and the effectiveness of education versus treatment. Additionally, elected pregnancy terminations, which were more common in the early years of France's screening program, account for some of the observed reduction in rates of congenital infection, but they are now very rare. Nevertheless, the screening program in France has eliminated or reduced the severity of virtually all clinically significant, adverse consequences due to congenital toxoplasmosis [9], [17], [19], [73].

## Methods

To determine whether a universal screening program for congenital toxoplasmosis might be cost saving in the United States, a decision-analytic model using TreeAge Pro Suite 2011 software (TreeAge Software, Inc., Williamstown, MA, USA) was developed, following a methodology used by Douglas Scott and colleagues at the US Centers for Disease Control and Prevention in Atlanta, Georgia [61] and by others to evaluate a newborn screening program [74]–[76]. The scope of the present study encompasses the lifetime consequences of congenital toxoplasmosis in the United States. The recipient population includes mothers and children screened and treated for congenital toxoplasmosis. Screening takes place in hospitals and clinical offices, while confirmation of any positive test result is performed at specialist reference laboratories with high standards of accuracy, such as the Toxoplasma Serology Laboratory at Palo Alto, California. The options considered are: (1) no systematic maternal screening and (2) universal maternal screening. The consequences of each option are limited to the various possible medical outcomes, relating to disease severity and treatment efficacy.

The decision model is structured to reflect the treatment protocol employed in the French (Parisian) congenital toxoplasmosis prevention program and the recommended approach for the management of toxoplasmosis during pregnancy [9], [56]. Accordingly, contrary decisions after the initial choice of option are not incorporated into the decision model because it is assumed that doctors will manage cases of congenital toxoplasmosis according to the recommended protocol.

The “no maternal screening” option assumes that no systematic screening is performed during pregnancy. Some doctors may recommend screening, generally if the mother lives with a cat. Cat-owning, however, is only one environmental risk for maternal infection. In the “no screening” arm, children who present with clinical congenital toxoplasmosis at birth are treated in the manner prescribed to the extent that their symptoms are accurately and promptly recognized. Outcomes in the no-screening arm include unrecognized disease, misdiagnosis, delayed diagnosis, delayed treatment, and consequent irreversible damage at birth or later for some children.

### Probabilities of Maternal Infection, Fetal Infection, Degree of Disease, and Other Variables

A full listing of probabilities and references is given in Table 1. The probabilities representing the efficacy of treatment at reducing adverse disease outcomes were derived primarily from Parisian data gathered at the reference center in Paris, France [15]–[22]. The percentages of children with disease manifestations reflect lifetime symptoms, not solely those that present at birth and are derived from clinical data and published studies [13], [57], [69], [77]–[80]. Probabilities reflecting prevalence of toxoplasmosis, primary infection in pregnancy, and incidence of CT in the United States were extrapolated from national and regional studies within the United States [45]–[50]. Additional documentation of the probabilities is contained in the file, Text S1, Supporting Information for Decision Tree and Table of Probabilities, available on line.

**Table 1:** Probabilities.

Phase	Variable	Point est. (range)	Reference(s)
*No Screening*	Prob primary infection in pregnancy	0.0011 (0.0004-0.0018)	[25], [26], [45], [50], [57], [68], [101]
	Prob fetal infection	0.50	[13], [57], [69], [77]–[79]
	Prob fetal death due to CT	0.05	[80]
	Prob no fetal disease	0.06	[57], [69], [77]
	Prob visual impairment	0.48	[57], [69]
	Of which mild	0.09	[57], [69]
	Prob visual and cognitive impairment	0.45 (0.40-0.55)	[57], [69], [77]
	Of which mild	0.39 (0.33-0.45)	[57],[69],[77]
	Prob visual, cognitive, hearing impairment	0.01	[57], [69], [77]
*12 Weeks*	Prob IgG(+) (maternal seroprevalence)	0.11	[26], [48], [50], [68], [101]
	Prob IgG(+) IgM(+)	0.0011 (0.0004-0.0018)	[26], [48], [50], [68], [101]
	Prob IgG(+) IgM(+) on confirmation	0.9	[26], [48], [50], [68], [101]
	Prob fetal death due to CT	0.02	[80]
	Prob fetal death due to amniocentesis	0.0025 (0.0006-0.0033)	[90], [92]
	Prob amniocentesis (–)	0.9635	[57], [67], [69], [78]
	Prob amniocentesis (+)	0.034	[57], [67], [69], [78]
	Prob no disease	0.6	[15]–[22], [102]
	Prob visual impairment	0.3 (0.10-0.40)	[15]–[22], [102]
	Prob visual and cognitive impairment	0.095	[15]–[22], [102]
	Prob visual, cognitive, hearing impairment	0.005	[15]–[22], [102]
*16 Weeks*	Prob IgG(+) (primary infection in pregnancy)	0.0011 (0.0004-0.0018)	[26], [48], [50], [68], [101]
	Prob IgG(+) IgM(+) on confirmation	0.9	[26], [48], [50], [68], [101], [103]
	Prob fetal death due to amniocentesis	0.0025	[90], [92]
	Prob amniocentesis (–)	0.9045	[57], [69], [73]
	Prob amniocentesis (+)	0.093	[57], [69]
	Prob no disease	0.85	[15]–[22], [102]
	Prob visual impairment	0.10	[15]–[22], [102]
	Prob visual and cognitive impairment	0.025	[15]–[22], [102]
	Prob visual, cognitive, hearing impairment	0.025	[15]–[22], [102]
*20 Weeks*	Prob IgG(+) (primary infection in pregnancy)	0.0011 (0.0004-0.0018)	[26], [48], [50], [68], [101]
	Prob IgG(+) IgM(+) on confirmation	0.9	[26], [48], [50], [68], [101], [103]
	Prob fetal death due to amniocentesis	0.0025	[90], [92]
	Prob amniocentesis (–)	0.8275	[57], [69], [73]
	Prob amniocentesis (+)	0.17	[57], [69]
	Prob no disease	0.85	[15]–[22], [102]
	Prob visual impairment	0.10	[15]–[22], [102]
	Prob visual and cognitive impairment	0.025	[15]–[22], [102]
	Prob visual, cognitive, hearing impairment	0.025	[15]–[22], [102]
*24 Weeks*	Prob IgG(+) (primary infection in pregnancy)	0.0011 (0.0004-0.0018)	[26], [48], [50], [68], [101]
	Prob IgG(+) IgM(+) on confirmation	0.9	[26], [48], [50], [68], [101], [103]
	Prob fetal death due to amniocentesis	0.0025	[90], [92]
	Prob amniocentesis (–)	0.7575	[57], [69], [73]
	Prob amniocentesis (+)	0.24	[57], [69]
	Prob no disease	0.85	[15]–[22], [102]
	Prob visual impairment	0.10	[15]–[22], [102]
	Prob visual and cognitive impairment	0.025	[15]–[22], [102]
	Prob visual, cognitive, hearing impairment	0.025	[15]–[22], [102]
*28 Weeks*	Prob IgG(+) (primary infection in pregnancy)	0.0011 (0.0004-0.0018)	[26], [48], [50], [68], [101]
	Prob IgG(+) IgM(+) on confirmation	0.9	[26], [48], [50], [68], [101], [103]
	Prob fetal death due to amniocentesis	0.0025	[90], [92]
	Prob amniocentesis (–)	0.7275	[57], [69], [73]
	Prob amniocentesis (+)	0.27	[57], [69]
	Prob no disease	0.94	[15]–[22], [102]
	Prob visual impairment	0.05	[15]–[22], [102]
	Prob visual and cognitive impairment	0.005	[15]–[22], [102]
	Prob visual, cognitive, hearing impairment	0.005	[15]–[22], [102]
*32 Weeks*	Prob IgG(+) (primary infection in pregnancy)	0.0011 (0.0004-0.0018)	[26], [48], [50], [68], [101]
	Prob IgG(+) IgM(+) on confirmation	0.9	[26], [48], [50], [68], [101], [103]
	Prob fetal death due to amniocentesis	0.0025	[90], [92]
	Prob amniocentesis (–)	0.3775	[57], [69], [73]
	Prob amniocentesis (+)	0.62	[57], [69]
	Prob no disease	0.94	[15]–[22], [102]
	Prob visual impairment	0.05	[15]–[22], [102]
	Prob visual and cognitive impairment	0.005	[15]–[22], [102]
	Prob visual, cognitive, hearing impairment	0.005	[15]–[22], [102]
*36 Weeks*	Prob IgG(+) (primary infection in pregnancy)	0.0011 (0.0004-0.0018)	[26], [48], [50], [68], [101]
	Prob IgG(+) IgM(+) on confirmation	0.9	[26], [48], [50], [68], [101], [103]
	Prob no disease	0.94	[15]–[22], [102]
	Prob visual impairment	0.05	[15]–[22], [102]
	Prob visual and cognitive impairment	0.005	[15]–[22], [102]
	Prob visual, cognitive, hearing impairment	0.005	[15]–[22], [102]
*Newborn*	Prob newborn test (+)	0.00055 (0.0002-0.0009)	[26], [48], [50], [68], [101]
	Prob IgG(+) IgM(+) on confirmation	0.9	[26], [48], [50], [68], [101], [103]
	Prob no disease	0.94	[15]–[22], [102]
	Prob visual impairment	0.05	[15]–[22], [102]
	Prob visual and cognitive impairment	0.005	[15]–[22], [102]
	Prob visual, cognitive, hearing impairment	0.005	[15]–[22], [102]
*Postnatal*	Prob postnatal test(+)	0.00055 (0.0002-0.0009)	[26], [48], [50], [68], [101]
	Prob IgG(+) IgM(+) on confirmation	0.9	[26], [48], [50], [68], [101], [103]
	Prob no disease	0.94	[15]–[22], [102]
	Prob visual impairment	0.05	[15]–[22], [102]
	Prob visual and cognitive impairment	0.005	[15]–[22], [102]
	Prob visual, cognitive, hearing impairment	0.005	[15]–[22], [102]

### Estimates of Costs of Injury

The perspective of the study is that of societal costs. Cost estimates, the payoffs in the decision tree framework, were derived from Research Triangle Institute's (RTI) report, *The Cost of Developmental Disabilities* [81]. The study used the cost-of-illness (COI) approach to assess the lifetime costs of five developmental disabilities (DD), three of which are relevant to congenital toxoplasmosis: severe cognitive impairment, hearing impairment, and visual impairment. By estimating social costs, the value of all resources used or lost as a result of the DD is included in the economic analysis, such as the costs of the medical and nonmedical services and equipment used as a result of the DD, as well as the earning and productivity losses for the infected persons and families who take time to care for the individual with a DD. The costs are incidence-based estimates, which measure the lifetime costs for an individual from the onset of the DD to death. Such estimates attempt to proxy potential cost savings that can be achieved through treatment to prevent or mitigate injury. Table 2 gives the cost estimates for various outcomes of congenital infection, with costs discounted at 3%, which is the recommended discount rate for health interventions used by the World Bank and the World Health Organization in the Global Burden of Disease reports [82]–[84].

**Table 2:** Cost estimates for outcomes of congenital toxoplasmosis (RTI estimates adjusted to 2010$).

Developmental Disorder	Costs (2010$) at 3% Discount Rate
Fetal Death (any cause)	$6,957,043
Visual, mild	$537,187
Visual, severe	$962,003
Cognitive, mild	$1,109,776
Cognitive, severe	$2,732,816
Hearing, mild	$383,635
Visual + Cognitive, mild	$1,646,963
Visual + Cognitive, severe	$2,910,003
Visual + Cognitive + Hearing, mild	$2,030,598
Visual + Cognitive + Hearing, severe	$3,293,638

Based on costs in Honeycutt A, Dunlap, L., Chen, H., al Homsi, G. (2000) The Cost of Developmental Disabilities: Task Order No. 0621-09. Research Triangle Institute [81] and US Bureau of Labor Statistics (http://data.bls.gov/cgi-bin/cpicalc.pl).

We include cost estimates for severe disease requiring home care, while mild disease does not require home care. For outcomes characterized by multiple disabilities of a mild nature, specific DD costs were summed to produce the cost estimate. For outcomes characterized by multiple disabilities of a severe nature, the cost estimate for severe cognitive damage was summed with the cost estimate(s) for the other individual disabilities without home care costs, to avoid double counting. Costs were calculated based on normal life expectancy of 76 years and adjusted for impairment-specific survival probabilities [81]. The outcomes of congenital toxoplasmosis listed in Table 2 were selected based on observed clinical outcomes, as confirmed through the medical literature [13], [57], [69], [77]–[80] and personal clinical experience (RM).

Indirect costs of psychological impacts borne by family members were not included in the estimates, nor were the costs associated with institutionalization in long-term care facilities. Mild cases are likely to have been underreported [81]. In sum, the assumptions made and limitations in the data are likely to bias the cost estimates downward, yielding a lower-bound estimate of the costs of each DD. Accordingly, the savings associated with prevention or mitigation of each disease outcome are likely to be higher than our estimates. We based the cost of fetal death on the range of estimates for the value of statistical life in the literature ($5,000,000) adjusted to October 2010 dollars [85]–[89]. This value is assigned to all fetal death outcomes, regardless of direct cause (disease or amniocentesis).

### Estimates of Screening and Treatment Costs

Total cost estimates of each disease outcome also include test and treatment costs incurred throughout gestation and one year of postnatal treatment, as shown in Table 3. At current volumes, serological tests for toxoplasmosis, including IgM, are priced at $12 per test. The Toxoplasmosis panel at a reference lab for confirmation of recent infection costs $385 (PAMF-TSL, http://www.pamf.org/serology). Amniocentesis is assigned a cost of $1300 per procedure (Personal communication to RM, M.Christmas M.D., Little Company of Mary Hospital, Chicago, 2011). The total dollar values for test costs included in each cost estimate reflect the cost per test multiplied by the number of tests required throughout pregnancy.

**Table 3:** Test costs.

Category	Test	Cost
*Screening*	Serological test for IgG, IgM	$12.00
	*Toxoplasmosis* serological profile (TSP)	$385.00
	Amniocentesis (PCR)	$1,300.00
*Blood Work*	Complete blood count	$10.00
*Treatment*	Spiramycin	$0.00
	Pyrimethamine	$1.56 per day
	Sulfadiazine	$12.48 per day
	Folinic Acid	$0.10 per day
	One-year pediatric PSF treatment	$210.00
	Drug compounding	$20.00 per week

*Source*: (http://www.ipharma.com, 2011).

Spiramycin is currently not commercially available in the United States. It can be obtained at no cost after consultation with the US Food and Drug Administration through a program with the pharmaceutical company, Sanofi-Aventis; the marginal cost to the firm is negligible because the drug is produced for other uses.

Pyrimethamine, sulfadiazine, and folinic acid are used to treat fetal infection directly for the duration of pregnancy starting after the 18^th^ week. At current output, pyrimethamine costs $1.56 per day and sulfadiazine costs $12.48 per day. Folinic acid costs $0.70 per week in parenteral formula administered orally. Treatment with PSF continues for approximately one year after birth, and costs for medicines total $210 for the entire year plus compounding cost of $20 to $50 per week (the lower value of $20 was used). In addition, there are twice-weekly complete blood counts for the mother for the duration of pregnancy and for the baby for the first year of life, at $10 per sample (ipharma.com, 2011).

**Figure 1:**
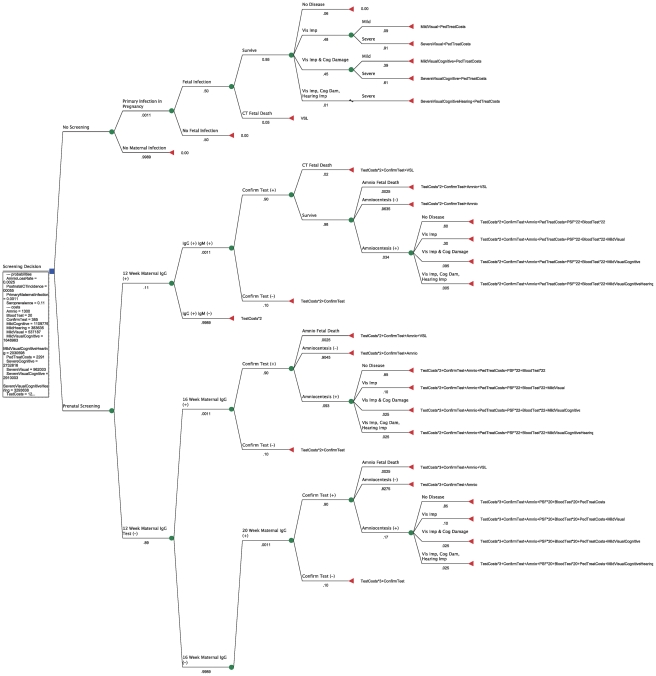
Decision tree with formulae (top).

**Figure 2:**
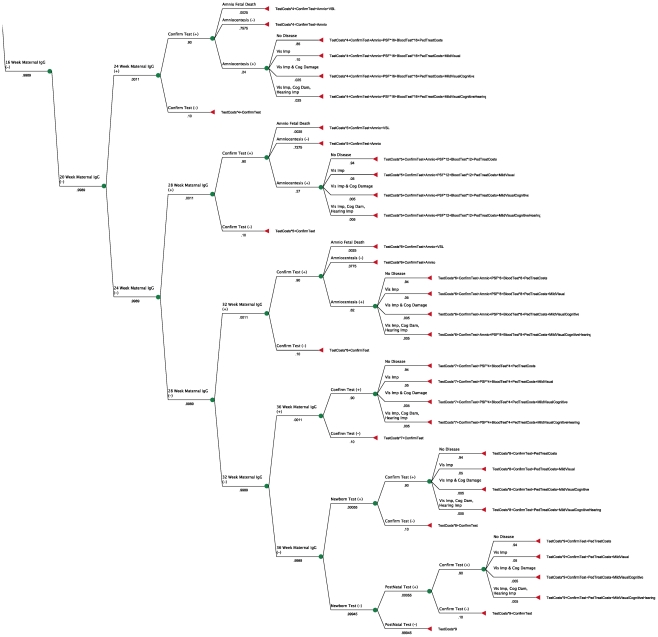
Decision tree with formulae (bottom).

**Figure 3:**
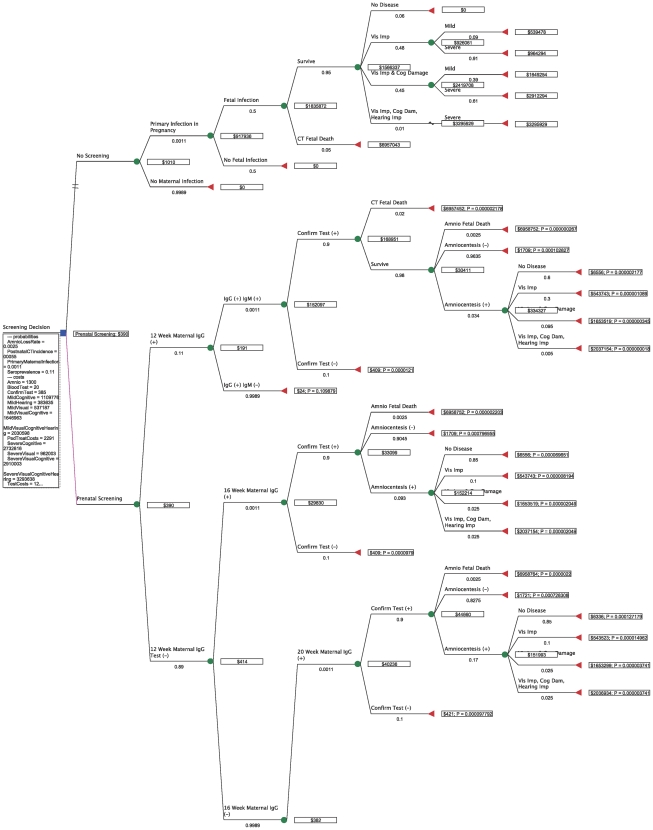
Decision tree with monetary values for costs and optimal path (top).

**Figure 4:**
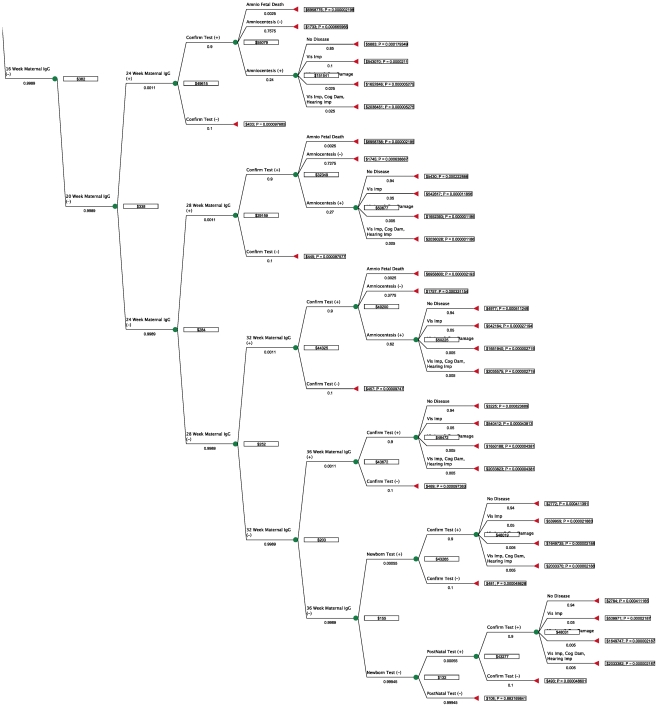
Decision tree with monetary values for costs and optimal path (bottom).

Total costs for each possible outcome are observable on the decision tree, Figures 1 and 2, which represent one tree divided to make it readable. Total cost estimates, reflecting test and treatment costs as well as estimated costs of disease, appear at each terminal node (denoted by a triangle), shown as formulae for the sum of each type of cost times its respective repetitions. As an example, a child born with mild visual impairment whose mother was tested at 12 weeks of gestation with a positive result and who transmitted to the fetus, in spite of spiramycin treatment, and was treated with PSF will entail costs as follows:

2 maternal tests + confirmatory test (Toxo panel at a reference lab) + amniocentesis + spiramycin (free) + PSF for 22 weeks (full term minus minimum age for PSF of 18 weeks) + 22 weeks of twice-weekly blood tests for mother + pediatric treatment for 52 weeks (including blood tests and compounding costs) + societal costs of mild visual impairment. Figures 3 and 4 show the total costs for each scenario in dollars and the probability of each outcome, as well as the optimal (cost-minimizing) path.

### Sensitivity Analysis

To determine the robustness of results to key parameters, sensitivity analysis was performed on incidence of primary *T. gondii* infection during pregnancy, population seroprevalence, risk of amniocentesis, the value of statistical life, and test costs because those variables would have the largest effect on model outcomes. Moreover, they are the variables most likely to vary between populations and thus warrant attention in applying the model to different populations. Net cost is particularly sensitive to seroprevalence because in populations with very low prevalence more mothers must be tested repeatedly throughout pregnancy. Similarly, the societal cost of CT is sensitive in low-incidence populations. The ranges for prevalence and incidence of primary infection during pregnancy were derived from estimates for the United States and regional surveys [13], [57], [69], [77]–[80].

Risk of fetal death from amniocentesis could have a significant impact on societal cost because the full value of statistical life is applied to fetal death. The range for risk of amniocentesis is derived from high and low estimates from CDC and other published sources [90]–[92]. The range for value of statistical life was derived from a search of estimates in the literature [86], [93]. Especially in low-prevalence populations, test costs could have a large impact on total cost. The upper bound derives from actual costs, and the lower bound is based on the cost of point-of-service tests for other conditions because most of the cost for testing is shipping and administrative expense.

Severity of untreated infection seems to vary between populations, suggesting that the South American strain is more virulent than the European strain [27]. Efficacy of treatment, however, appears to be similar between populations [94]. For the US population, therefore, sensitivity analysis is not warranted for efficacy, the value of which is established in the literature [14], [16]–[18], [20], [21], [56], [66].

## Results and Discussion

Our results indicate that universal screening is cost saving at an expected cost of $390 per child screened, inclusive of societal cost of remaining DD, compared to an expected societal cost of congenital toxoplasmosis of $1010 per birth under the “no maternal screening” alternative. Thus, cost savings of $620 per child are predicted with the implementation of a universal maternal screening program in the US population with an estimated 4 million births per year, or nearly $2.5 billion saved annually compared to no maternal screening. Accordingly, the initial model suggests a policy recommendation on the basis of cost savings in favor of a universal maternal screening program for congenital toxoplasmosis following that currently employed in France. The results were robust to changing the discount rate to 5%, although the expected saving was reduced to $371 per child screened. Eliminating amniocentesis at 32 weeks had no significant effect on the results.

### Sensitivity Analysis

**Figure 5:**
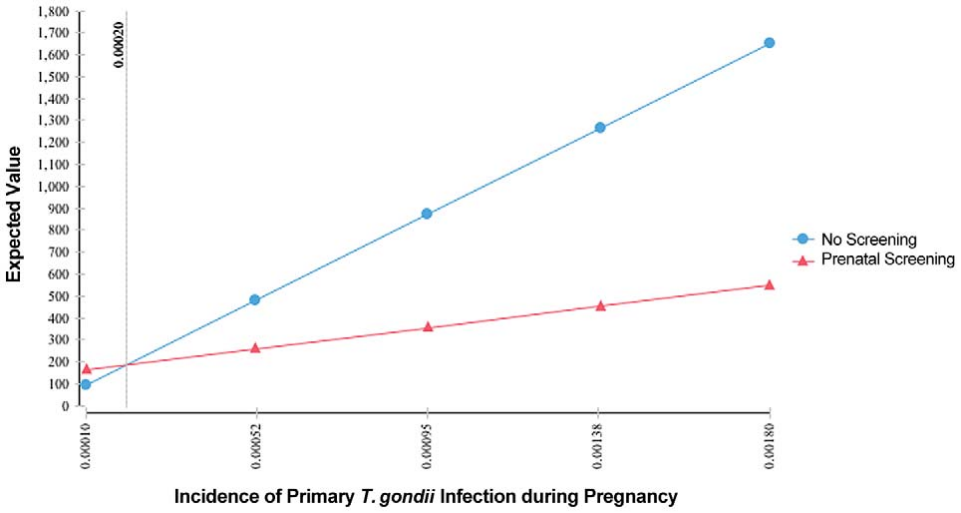
Sensitivity analysis of incidence of primary *T. gondii* infection during pregnancy.

**Figure 6:**
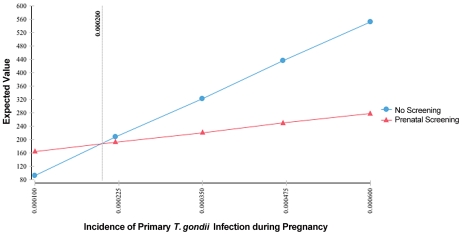
Sensitivity analysis of incidence of primary *T. gondii* infection during pregnancy (detail).

**Figure 7:**
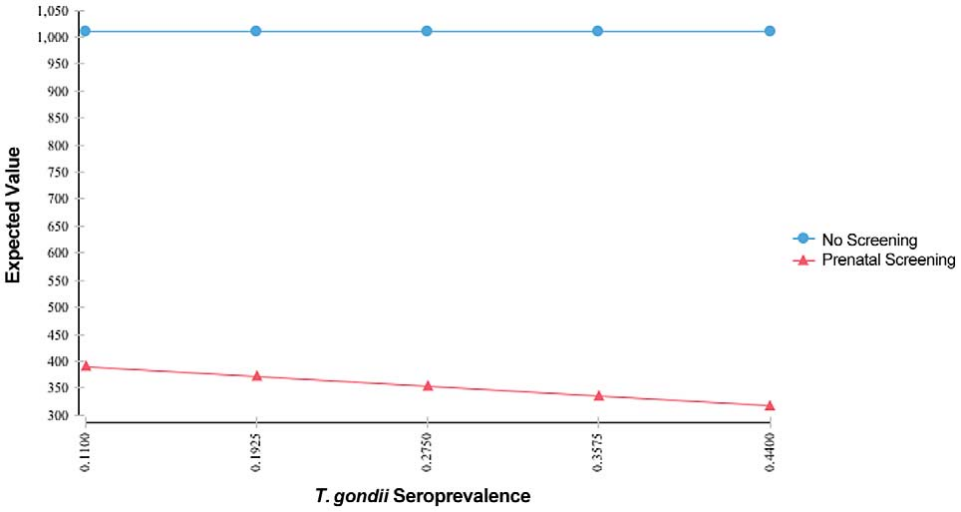
Sensitivity analysis on *T. gondii* seroprevalence.

**Figure 8:**
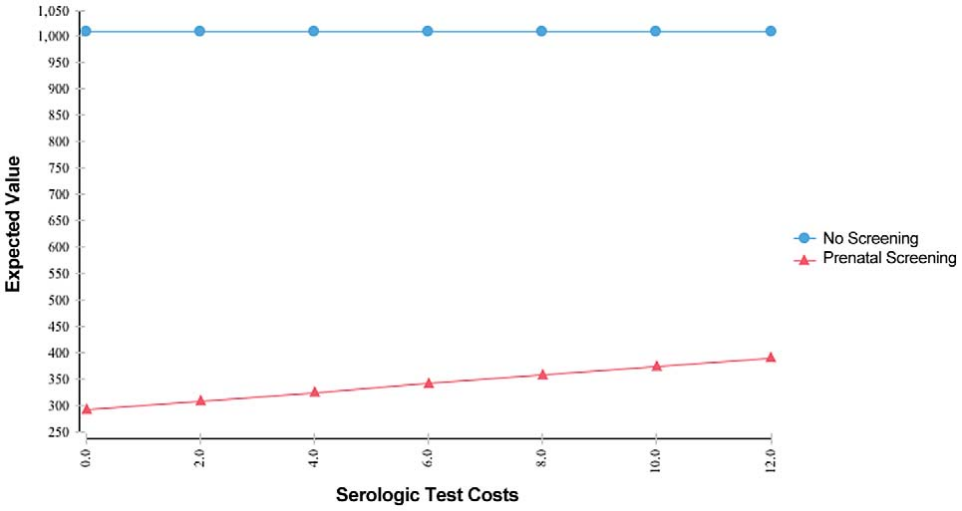
Sensitivity analysis on serologic test costs.

One-way sensitivity analysis graphs appear in Figures 5, 6, 7, and 8. The two lines on each graph correspond to the relevant decision (screening versus no screening), and any deviation from horizontal reflects that strategy's sensitivity to the variable under consideration. In our model, only differences in the incidence of primary infection during pregnancy produce a change in the optimal strategy, which occurs below the lower bound of estimates for the United States. As seen in Figure 5 (enlarged in Figure 6), for rates of primary infection during pregnancy of less than 0.0002, universal screening becomes the non-optimal strategy. Maternal incidence of 0.0002 corresponds to incidence of congenital infection on average of 0.0001 (1 in 10,000), based on the 0.50 probability of maternal transmission (over all trimesters) without treatment [13], [57], [69], [77]–[79]. As a result, in populations with extremely low rates of congenital infection, maternal screening is not found to be cost-saving. For other reasons, one might conclude that screening is the correct decision, but that determination is beyond the focus of this study.

For all other variables tested, variation over the specified range of values reveals that the screening strategy is sensitive to those assumptions, but no threshold values are reached (Figures 7 and 8, and other analyses not shown). The expected value for cost of prenatal screening is less than the expected value for the cost of the no-screening option for all values of the variables tested. Cost savings increase with population seroprevalence (Figure 7) since populations with higher rates of *T. gondii* infection, over a broad range, have higher rates of seroconversion over childbearing years and thus higher benefits (lower societal cost of injury) from screening. High-prevalence populations also have a smaller pool of susceptible women and thus will have lower cost of testing. Conversely, cost savings from universal screening decline with increasing fetal loss rates due to amniocentesis, with increasing serological test costs (Figure 8), and with cost of amniocentesis, but screening remains dominant. Lastly, variation of the value of statistical life from $600,000 to $10,000,000 had an equal effect on both the screening and no screening strategies.

**Figure 9:**
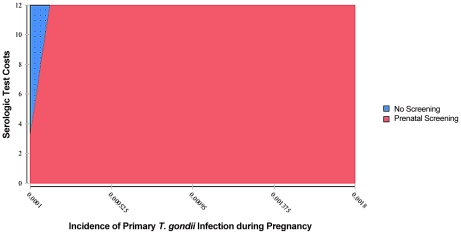
Two-way sensitivity analysis on incidence of primary *T. gondii* infection during pregnancy and serologic test costs.

**Figure 10:**
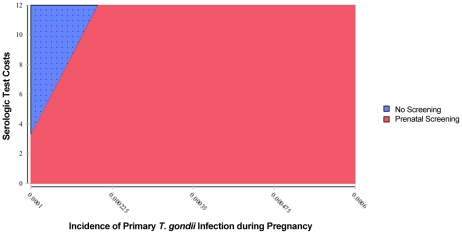
Two-way sensitivity analysis on incidence of primary *T. gondii* infection during pregnancy and serologic test costs (detail).

Two-way sensitivity analysis was performed on the variables for test costs and the rate of primary infection during pregnancy. Figures 9 and 10 reveal that reducing test costs effectively lowers the rate of primary infection in pregnancy for which screening is cost saving. Notably, if test costs are lowered significantly (to roughly $2 per test, which is feasible given the cost of other in-office diagnostic tests), screening becomes the optimal strategy even at rates of primary infection in pregnancy well below the lowest reported rates of 2 in 10,000 in the United States.

### Discussion

The decision-analytic model developed in this paper reveals that for populations with rates of congenital toxoplasmosis greater than 0.0001 (1 infected child per 10,000 live births, or 2 infected mothers per 10,000), maternal serological screening is a cost-saving strategy. This finding is robust to changes in seroprevalence, incidence of maternal primary infection, amniocentesis risk, value of statistical life, and test costs. Given current estimates of the rate of congenital infection in the United States, implementation of a universal screening program for congenital toxoplasmosis prevention and treatment is predicted to generate cost savings of approximately $620 per birth.

Sensitivity analysis shows that even for populations with extremely low rates of congenital infection, screening is cost saving at a test cost of $12 plus confirmatory test at $385. Nevertheless, policy makers must be cautious when considering estimates of rates of congenital infection. Some studies have found rates of congenital infection in the United States below 0.0001 [49], [50], casting doubt on cost saving by universal screening as an intervention strategy at current test costs, although with our estimate of 0.0011 for the rate of primary infection in pregnancy, based on the midpoint of estimates from available sources, screening is a cost-saving strategy.

Furthermore, if screening is initiated, we expect to observe economies of scale in test production. If test costs are reduced, screening becomes cost saving even in populations with rates of congenital infection below 0.0001. Accordingly, as universal screening is enacted and test production expanded, economies of scale in test production may render screening a cost-saving strategy for all populations, even at extremely low rates of congenital infection. The capacity to test for several congenital conditions while drawing blood at one time opens the possibility of other cost savings (economies of scope). Pooling the costs of testing for congenital cytomegalovirus and other conditions, for example, would reduce the threshold for cost savings for all conditions.

The cost-minimization analysis described herein demonstrates that a careful and robust gestational screening program as carried out in France can be a cost-saving intervention in the United States. This paradigm can be readily applied to evaluating options for preventing toxoplasmosis in developing countries worldwide. In Brazil, for example, carefully performed and detailed regional programs are collecting data concerning gestational infection and congenital toxoplasmosis, which are amenable to analysis with the paradigm developed herein [27]. This paradigm is also readily applicable to analyses of other neglected tropical diseases.

### Limitations

The recommendation of screening is complicated by the disparity between a best practice scenario, such as that analyzed in this study, and US health care reality. The above analysis implicitly assumes that all mothers receive care by the twelfth week of gestation for monthly checkups and adhere to the advice of their primary care providers in decisions regarding the management of pregnancy. In actuality, this scenario is unlikely. Lower-income mothers may lack the resources to travel to monthly checkups or may be discouraged from visits by a lack of health insurance or poor access to, or poor treatment in, public facilities. This study viewed all costs from a societal perspective and thus abstracted from the incidence of test and treatment costs. Even if the costs are covered by insurance, mothers without insurance will have a disincentive to report for pregnancy checkups. If this is the case, the benefits to be derived from screening for congenital toxoplasmosis will not be evenly distributed across income strata and demographics, and societal benefits are correspondingly reduced as well. This analysis assumes that initiating best practice is an essential step to promoting adherence and will contribute to the momentum for universal access to health care, in particular adequate prenatal care, which provides other benefits to society as well.

Adherence can also be impaired due to maternal preference, regardless of income or accessibility. In France, in spite of compulsory universal screening, public medical care was associated with a late first test, fewer tests, and longer intervals between tests [95]. Public health coverage in the United States would need to address those shortcomings to achieve universal screening. Policy makers will also have to consider not only the extent to which screening may be cost saving, but also the important question of “who pays?” Taxpayers will likely bear the burden of test and treatment costs for low-income mothers.

A further consideration is necessitated by the potentially low positive predictive value of maternal serum tests. If specificity (identification of true negatives) is less than 100%, the low prevalence of congenital infection dominates the calculation of positive predictive value, resulting in tests for which as few as 20% of positive test results correspond to actual infection.

The present study is based on the assumption of 100% specificity after confirmatory test at a high quality reference lab. A study sponsored by the US Centers for Disease Control and Prevention (CDC) compared six test kits available for the detection of *T. gondii* antibodies in serum and found the sensitivity of the tests that might be used for screening ranged from 93.3% to 100% and the specificity ranged from 77.5% to 99.1% [96]. A more recent study compared the performance of four different *Toxoplasma* IgG and IgM assays. The Toxo assays considered were Vidia Toxo IgG and IgM (bioMerieux, Marcy l'Etoile, France), Vidas Toxo IgG and IgM (bioMerieux), AxSYM Toxo IgG and IgM (Abbot Laboratories, Abbot Park, IL), and Liaison Toxo IgG and IgM (Dia-Soring, Saluggia, Italy). For the *Toxoplasma* IgG assays, sensitivity was 100% and specificity between 98.49% and 100%. The *Toxoplasma* IgM assays performed with sensitivity between 82.35% and 100% and specificity between 99.73% and 100% [97].

For diseases with very low prevalence, even very high (but less than 100%) specificity translates into low positive predictive value (PPV), which is the probability that disease is truly present given that the result of the screening test is positive. Written Pr(D+|T+), it is the posterior probability of a true-positive test result. Mittendorf and colleagues demonstrate clearly the effect of less than 100% specificity on PPV and argue that routine screening for toxoplasmosis in the United States is unwarranted because of the low incidence of congenital infection. Furthermore, due to the calculated low positive predictive value of serology tests for toxoplasmosis, they estimated that 12.1 fetuses without CT would be aborted for every fetus detected with congenital toxoplasmosis [98]. Given these calculations, they concluded that the adverse effects for healthy fetuses of universal screening outweigh the benefits derived from early detection and treatment of infected fetuses.

The implications of the Mittendorf et al. analysis were considered very carefully. There are two reasons that their analysis does not apply. First, the protocol herein requires a confirmatory test at a reference lab, which at the present time has a specificity of 100%; second, our study calculates cost based on best practice. Mittendorf and colleagues and others using their analysis postulate elected abortions upon a positive confirmatory test [64], [98], [99]. CT is a treatable condition in almost all cases and thus, treatment, not abortion, is almost always considered best practice. Therefore, our model calculates the cost savings without assuming elected abortions. In recent years in France, there have been so few elected terminations (three reported in 2008) [9], [17] that including estimates would not alter our results. For infected mothers, the adverse consequences of universal screening can be reduced through the use of further confirmatory testing, such as amniocentesis with PCR. A recent study of the use of PCR of amniotic fluid in France reported a specificity of 100%, sensitivity of 92%, and positive predictive value of 100% [14], [73], meaning that a positive result definitively identifies infection of the fetus [73].

The use of amniocentesis to confirm fetal infection (positive predictive value of 100%) can reduce the risk of misinformed abortion, but this potentiality hinges on proper education of primary care physicians and mothers. The potential problems posed by positive predictive value are not devastating to the implementation of a screening program, although they do urge caution and diligence in the implementation of any such programs.

While the use of PCR of amniotic fluid may prevent unnecessary elected abortions of healthy fetuses, sampling amniotic fluid is an invasive procedure and itself carries a risk of fetal loss. In the 1970s, the risk of miscarriage due to amniocentesis was estimated to be 0.50% (1 in 200) [91]; but in that era continuous ultrasound guidance for the procedure was not routine, and clarity and quality were far inferior to that of ultrasonography today. Amniocentesis is now considered routine, where medically indicated, and recent studies suggest a far lower rate of fetal loss due to amniocentesis; the Mayo Clinic reports loss rates of 1 in 300 (0.33%) to 1 in 500 (0.20%), and a recent study suggests far lower loss rates of 1 in 1,600 (0.06%) [90], [92]. We use an average of the two Mayo Clinic estimates in the model (0.25%), which may greatly overstate risk.

A concern with adding any testing to a prenatal protocol is the additional anxiety for mothers. Serology for *T. gondii*, however, can be carried out along with other routine tests and need not impose unusual stress. Moreover, mothers need not be informed of suspicious results until the sample has been confirmed with a second test and, if positive, sent for further testing to a reference lab. Thus only true positives would be informed of the need for medication and amniocentesis. Certainly, those both generate concern and risk. Except in extreme cases, however, mothers are not anxious about impossible outcomes, but about real outcomes, albeit having low probability in most cases. Awareness of personal agency is a powerful antidote to anxiety. Knowledge that they can help their unborn children with better information would be more likely to alleviate maternal anxiety than to make it less bearable.

An additional limitation of the model is that only two possible strategies were analyzed: no screening and prenatal screening and treatment. Pre-pregnancy education was incorporated into the model via sensitivity analysis of reduction in seroprevalence and primary infection during pregnancy to assess the impact of the efficacy of education on the decision. Reducing the rate of maternal and congenital infection by as much as 60% (the suggested effect of maternal education) reduces the extent to which screening is cost saving, although, even with that effect, subsequent screening remained the optimal strategy. Recent analyses indicate that many times risk factors for *T. gondii* infection go unrecognized and thus could not be eliminated by education alone [29], [59], [100]. Additionally, universal neonatal screening was not considered in the model. Incorporation of this third strategy could render prenatal screening a sub-optimal strategy if cost is the only consideration, although screening would still be cost saving when compared to no systematic screening and no screening at all. Neonatal screening, however, misses the opportunity to treat prenatally and prevent profound injury with life-long consequences for the child, the family, and society.

Finally, sensitivity analysis on the efficacy of treatment was not performed. Treatment efficacy estimates were generated based on published clinical results, and thus have not been subjected to sensitivity analysis, although variation of these estimates could have a potentially significant effect on the results generated by the model. More extensive screening and treatment in the United States would contribute to knowledge of efficacy in this population, with its possible mixture of European and Western Hemisphere strains of *T. gondii*, although preliminary results suggest that treatment is equally efficacious for different strains [94]. Moreover, if congenital toxoplasmosis becomes a more widely understood and reported disease in the United States, estimates of the rate of congenital infection will become more accurate and region-specific. A protocol of screening in suspected high-incidence populations would be another alternative.

### Conclusion

Universal screening according to the French protocol is cost saving for the US population within broad parameters for costs, seroprevalence, incidence of maternal and congenital toxoplasmosis, value of statistical life, and risk of fetal death from amniocentesis. It is also robust to changes in the discount rate within the normal range for health interventions.

## Supporting Information

Text S1 **Supporting information for decision tree and table of probabilities.**
(PDF)
