# Supplementary material for: Maternal Serologic Screening to Prevent Congenital Toxoplasmosis: A Decision-Analytic Economic Model
Source: PLoS Negl Trop Dis. 2011 Sep 27;5(9):e1333. doi: 10.1371/journal.pntd.0001333 (PMC3181241; doi:10.1371/journal.pntd.0001333)
Supplement: Text S1 — Supporting information for decision tree and table of probabilities. (PDF) [file pntd.0001333.s001.pdf]

Supporting Information for Decision Tree and Table of Probabilities

This document is intended to provide detailed background information and justification for the probabilities used in the decision-analytic economic model. All probabilities used, as well as their references, may be found in **Table 1: Probabilities, presented in the article**. After a brief discussion of background information, each probability is given and supporting information provided. The "No Screening" scenario is addressed first, followed by the "Screening" scenario.

Background Information:

The Parisian data were derived from the reference center in Paris, France in the context of government-mandated, systematic screening programs. Screening during gestation began in France in 1978 and monthly screening was implemented in 1992. Treatment with spiramycin began in 1972. Pyrimethamine and Sulfadiazine treatment was initiated in 1983 in alternate months with spiramycin, and this approach continued through 1989. Continuous pyrimethamine and sulfadiazine treatment of pregnant women whose fetus had confirmed infection was begun in 1990s. Continuous pyrimethamine and sulfadiazine treatment of infants for one year after birth was begun in Paris in the early 1990s. Pooled data with different treatments in different centers were used only in the Paris, Lyon, Marseille data of Kieffer et al. 2008 [1]. Otherwise differences in approaches from different centers described in manuscripts with pooled data were not used for our determinations of percentages. The percentages for those with disease manifestations are those over a lifetime and not findings present solely at birth.

No Screening:

1. Probability of Primary *T. gondii* infection in pregnancy = 0.0011 (.0004-.0018)
Estimates are based on the following collections of data: ~1984-86 and ~1986-88, age-specific seroprevalence in pregnant women in two Chicago hospitals (The University of Chicago Hospitals and Michael Reese Hospital and Medical Center, Papoz mathematical model. (RM, personal communication, 2011). Similar modeling using the NHANES data 2000-2004 (RM, personal communication, 2011); Massachusetts screening program actual data [2]; data from Denmark indicate that this type of testing detects about 50% of infected babies; combined with 2, below. **Transmission rates from Tables 1, 2, and 3 of this document.**

2. Probability of Fetal Infection = 0.50 (range 36-61%)
If a mother acquires *T. gondii* in her pregnancy, her fetus is at risk of acquiring the parasite as well. The risk of transmission to the fetus over a pregnancy with no treatment interventions is approximately 50% (range 36-61%). This was determined by Desmonts, Couvreur, 1979, **Tables 1 and 2 herein.** Table 1 shows that the total number of positive isolations of *Toxoplasma* in placentas was 50%. Table 2 shows that the number of babies with no congenital infection is 39% with no treatment of mother. Thus, the percentage of babies with congenital infection is 61%. Table 3 from Desmonts G and Courvreur J, 1974 [3] shows a transmission incidence of 36%. From these data there appears to be a 36-61% risk of transmission in the absence of treatment; an intermediate value of 50% is used herein.

3. Probability of fetal death due to congenital toxoplasmosis (without treatment) = 0.05
In Berrebi et al, [4].

4. Probability of No Disease in Baby = 0.06
If a baby is infected, there is a very small chance of no disease ever being present in the child. Even those children with subclinical infection at birth are at risk for adverse sequelae. According to Wilson, et al, 1980, 86% of children with subclinical infection developed chorioretinal lesions with 81% of those developing unilateral blindness [5]. For the others who do not have subclinical infection like those in the Wilson et al study, they would have moderate or severe signs of infection at birth. **Please see Table 2 and Tables 4 to 9 and Figure 1.** These Tables and Figures are also relevant to other analyses.

5. Probability of Visual Impairment alone in Baby = 0.48
Even those children with subclinical infection at birth are at risk for adverse sequelae. According to Wilson, et al, 1980, 86% of children with subclinical infection developed chorioretinal lesions with 81% of those developing unilateral blindness[5]. Other children, not those with subclinical infection, have moderate or severe signs of infection at birth. About half of the untreated children with chorioretinal lesions have some neurological findings as well. **See Tables 2 to 8 and Figure 1.**

6. Probability of Visual Impairment and Cognitive Damage in Baby = 0.45
See explanation of 5 above. Incidence may even be higher if you note that Wilson et al, 1980 describe that 86% have decrease in IQ by age 9 years among those with subclinical infections at birth. Specifically, Desmonts and Couvreur and others described the severity of manifestations at birth and later in life with and without treatment. The percentage of individuals with subclinical involvement is about 55%. The percentage of those with mild involvement is approximately 34%, and those with severe involvement approximately 10%. In other studies of those with subclinical infection [6,7,8] between 82% and 86% had retinal disease by adolescence or by 10 years of age. Thus, 45% had manifestations already and 86% of the remaining 55% developed ophthalmologic disease by their teenage years. Wilson, et al indicate that 86% of children with subclinical infection at birth have chorioretinal lesions and 81% unilateral blindness, which indicates severe involvement of the retina. Of those children with severe and irreversible disease manifestations at birth, virtually all have severe eye disease. A proportion of those with mild manifestations of infection (i.e. peripheral retinal scars, calcifications, CSF Pleocytosis) at birth will progress to severe visual impairment. This increases the percentage from 81% to 91%. Ocular toxoplasmosis is a recurrent and progressive disease. **See Table 4**.

7. Probability of Visual Impairment, Cognitive Damage, and Hearing Impairment in Baby = 0.01
See explanations and tables above. Note: Frequency of hearing loss in Wilson et al and Eichenwald of 14% [9]. Recent study in Brazil 4% [10]. U.S. currently sensorineural hearing loss not seen.

8. Probability of Visual Impairment being Mild=0.09
According to Wilson, et al, 1980 [5], 86% of children with subclinical infection developed chorioretinal lesions with 81% of those developing unilateral blindness. Others, i.e., those not in the subclinical infection category, have moderate or severe signs of infection at birth. **See Table 4, 5, 6, 8, and Figure 1**.

9. Probability of Visual Impairment being Severe=0.91
According to Wilson, et al, 1980, 86% of children with subclinical infection developed chorioretinal lesions with 81% of those developing unilateral blindness. Others have moderate or severe signs of infection at birth including visual impairment (i.e., ~70% of those in the NCCCTS cohort had retinal damage at birth). **See Table 4**.

10. Probability of Visual Impairment and Cognitive Damage being Mild=0.39
According to Wilson, et al, 1980, of those with subclinical infection at birth, 33% will develop severe, permanent neurologic sequelae, 14% will be cognitively impaired, and 86% will have sequentially lower IQ scores. The others already have moderate or severe signs of infection at birth. **See Table 4, 6**.

11. Probability of Visual Impairment and Cognitive Damage being Severe=0.61
Please see explanation of 10, and **Tables 4, 6**.

With Screening:

12. Probability of 12 Week test being IgG+=0.11
With screening, the first test will pick up both chronically and acutely infected women. In the US, approximately 11% of women of childbearing age are IgG+.

13. Probability of 12 week test being IgG+ and IgM+ = 0.0011 (0.0004 -- 0.0018)
The probability of being IgG+ and IgM+ is the estimated seroconversion rate.

14. Probability of confirmatory testing being + = 0.90
The confirmatory tests are 100% sensitive. After first trimester test, when seroconversion takes place, they are also 100% specific. In the first trimester, a proportion of chronically infected women who retain IgM will not have the high avidity result and chronic AC/HS usually present in chronic infection. We have estimated this to be ~10%. In this case, dating infection to before conception will not be feasible.

15. Probability of Fetal Death from *T. gondii* = 0.02
Fetal death due to amniocentesis is 0.0025. Villena et al, 2010 report that fetal death due to *T. gondii* is approximately .0183, occurring largely in the first trimester. Thus, the total probability of fetal death at the 12 week point is .0025+.0183=.0208. Also in Berrebi [4].

16. Probability of fetal death from amniocentesis = 0.0025
Midpoint of estimates from [11,12].

17. Probability of negative amniocentesis=0.9635

18. Probability of positive amniocentesis=0.034
See Tables 10 and 11 for probabilities of transmission of infection where there has been prenatal diagnosis and treatment. Values were interpreted using these tables. Amniocentesis is 92% sensitive and 100% specific with q and PCR for the 300 copy 529 bp gene. This and all subsequent probabilities of positive amniocentesis have been calculated as follows:
Incidence Rate at specific week gestation X .92 (Sensitivity of Amniocentesis)

19–22 apply to infection detected at 12 weeks
19. Probability of baby having no disease = 0.60
See Tables 2, 5, 8

20. Probability of baby having Visual Impairment = 0.30
See Tables 2, 5, 8, and Figure 2.

21. Probability of baby having Visual and Cognitive involvement = 0.095

22. Probability of baby having Visual Impairment, Cognitive Damage and Hearing loss=0.005

23. Probability of negative amniocentesis=0.9045

24. Probability of positive amniocentesis=0.093
See Table 10 and 11.

25–28 apply to infection detected at 16 to 24 weeks
25. Probability of infected baby having no disease=0.85

26. Probability of infected baby having visual impairment=0.10
See Table 12.
Statement in reference Villena, et al, 2010 [13]: In France, spiramycin is prescribed upon a finding of seroconversion in pregnant women. All such women were treated with this antibiotic, although its impact on vertical transmission is still controversial. When amniocentesis is positive, spiramycin treatment is stopped and a pyrimethamine–sulfonamide combination is generally prescribed until delivery. This antibiotic combination is considered to be effective in reducing the risk of severe congenital sequelae.
See Figure 2.

27. Probability of baby having Visual Impairment and Cognitive = 0.025

28. Probability of baby having Visual Impairment, Cognitive Damage and Hearing loss=0.025

29. Probability of negative amniocentesis=0.8275

30. Probability of positive amniocentesis=0.17
See explanation and **Tables 10 and 11**. Amniocentesis is 92% sensitive.

31. Probability of negative amniocentesis = 0.7575

32. Probability of amniocentesis being + = 0.24

33. Probability of amniocentesis being - = 0.7275

34. Probability of amniocentesis being + = 0.27

35–38 apply to infection detected at 28 weeks to one month postnatal
35. Probability of infected baby having no disease=0.94
These estimates of injury (35–38) apply to detection of infection and treatment at 28 to 36 weeks of gestation and Newborn test and one-month Postnatal test.

36. Probability of infected baby having visual impairment=0.05
See above.

37. Probability of infected baby having visual impairment and cognitive damage=0.005

38. Probability of baby having Visual Impairment, Cognitive Damage and Hearing loss=0.005

39. Probability of amniocentesis being - = 0.3775

40. Probability of amniocentesis being + = 0.62

41. There is no amniocentesis at 36 weeks; assumption is that all positive confirmatory tests will be followed by treatment.

42. Probability newborn test + = 0.00055
Based on probability of primary infection in pregnancy above in 1 and 13. It is assumed that there is constant risk of primary infection for mothers over the duration of pregnancy, so risk of new infection in perinatal period is half of maternal rate (based on 2 above). This understates the risk in the perinatal period since risk of transmission from mother to child increases over the pregnancy. Thus, this is a lower bound estimate.

Sources

1. Kieffer F, Wallon M, Garcia P, Thulliez P, Peyron F, et al. (2008) Risk factors for retinochoroiditis during the first 2 years of life in infants with treated congenital toxoplasmosis. Pediatr Infect Dis J 27: 27-32.

2. Guerina NG, Hsu H, Meissner HC, Maguire JH, Lynfield R, et al. (1994) Neonatal serologic screening and early treatment for congenital toxoplasma gondii infection. New England Journal of Medicine 330: 1858-1863.
3. Desmonts G, Couvreur J (1974) Toxoplasmosis in pregnancy and its transmission to the fetus. Bulletin of the New York Academy of Medicine: Journal of Urban Health 50: 146-159.
4. Berrebi A, Bardou M, Bessieres M, Nowakowska D, Castagno R, et al. (2007) Outcome for children infected with congenital toxoplasmosis in the first trimester and with normal ultrasound findings: A study of 36 cases. European Journal of Obstetrics Gynecology and Reproductive Biology 135: 53-57.
5. Wilson CB, Remington JS, Stagno S, Reynolds DW (1980) Development of adverse sequelae in children born with subclinical congenital *Toxoplasma* infection. Pediatrics 66: 767-774.
6. Koppe JG, Loewer-Sieger DH, de Roever-Bonnet H (1986) Results of 20-year follow-up of congenital toxoplasmosis. Lancet 327: 254-256.
7. Koppe JG, Kloosterman GJ (1982) Congenital toxoplasmosis: long-term follow-up. Padiatr Padol 17: 171-179.
8. Stagno S, et al. (1977) Auditory and visual defects resulting from symptomatic and subclinical congenital cytomegaloviral and *Toxoplasma* infections. Pediatrics: 669-678.
9. Eichenwald HF (1960) A study of congenital toxoplasmosis, with particular emphasis on clinical manifestations, sequelae and therapy. In: Siim JC, editor. Human toxoplasmosis. Munksgaard, Copenhagen. pp. 41-49.
10. Andrade GM, Resende LM, Goulart EM, Siqueira AL, Vitor RW, et al. (2008) Hearing loss in congenital toxoplasmosis detected by newborn screening. Braz J Otorhinolaryngol 74: 21-28.
11. Mayo Clinic (2010) Amniocentesis. Mayo Clinic.
12. Eddleman KA, Malone FD, Sullivan L, Dukes K, Berkowitz RL, et al. (2006) Pregnancy Loss Rates After Midtrimester Amniocentesis. American College of Obstetricians and Gynecologists 1067-1072.
13. Villena I, Ancelle T, Delmas C, Garcia P, Brezin AP, et al. (2010) Congenital toxoplasmosis in France in 2007: first results from a national surveillance system. Euro Surveill 15: pii-19600.

Table 1: Contrasting transmission at birth with and without treatment with spiramycin: Attempts to isolate *Toxoplasma** from placenta at delivery in women who acquired *Toxoplasma* infection during pregnancy

Maternal Treatment During Pregnancy	Infection Acquired During First Trimester		Infection Acquired During Second Trimester		Infection Acquired During Third Trimester		Total	
	No. Examined	No. Positive (%)	No. Examined	No. Positive (%)	No. Examined	No. Positive (%)	No. Examined	No. Positive (%)
None	16	4 (25)	13	7 (54)	23	15 (65)	52	26 (50)
Spiramycin	89	7 (8)	144	28 (19)	36	16 (44)	269	51 (19)
Total	105	11 (10)	157	35 (22)	59	31 (53)	321	77 (24)

*By Mouse inoculation

Source: Adapted from Desmonts G, Couvreur J. Congenital toxoplasmosis: a prospective study of the offspring of 542 women who acquired toxoplasmosis during pregnancy: pathophysiology of congenital disease. In Remington JS, McLeod R, Thulliez P, Desmonts G (2010) Toxoplasmosis. In: Remington J, Klein G, Wilson C, Baker C, editors. Infectious Disease of the Fetus and Newborn Infant. 6th ed. ed. Philadelphia: W.B. Saunders. pp. 947-1091, with permission.

Table 2: Contrasting transmission and findings at birth with and without treatment with spiramycin: Outcome of 542 pregnancies in which maternal *Toxoplasma* infection was acquired during gestation: Incidence of Congenital Toxoplasmosis and effect of spiramycin treatment in mother during pregnancy

	No. of Affected Infants	
Outcome of Offspring	No Treatment	Treatment
No congenital *Toxoplasma* infection	60 (39)	297 (77)
Congenital toxoplasmosis		
Subclinical	64 (41)	65 (17)
Mild	14 (9)	13 (3)
Severe	7 (5)	10 (2)
Stillbirth or perinatal death*	9 (6)	3 (1)
Total	154 (100)	388 (100)

*See Reference.

Source: Adapted from Desmonts G, Couvreur J. Congenital toxoplasmosis: a prospective study of the offspring of 542 women who acquired toxoplasmosis during pregnancy: Pathophysiology of congenital disease. In Remington JS, McLeod R, Thulliez P, Desmonts G (2010) Toxoplasmosis. In: Remington J, Klein G, Wilson C, Baker C, editors. Infectious Disease of the Fetus and Newborn Infant. 6th ed. ed. Philadelphia: W.B. Saunders. pp. 947-1091, with permission.

Table 3: Incidence of Transmission and Severity of Disease in each Trimester

Trimester of Maternal Acquisition	Incidence of Transmission (%)	Relative Severity of Disease
I	17	Severe
II	25	Intermediate Severity
III	65	Milder or asymptomatic

Source: Adapted from Desmonts G, Couvreur J (1974) Toxoplasmosis in pregnancy and its transmission to the fetus. Bulletin of the New York Academy of Medicine: Journal of Urban Health 50: 146-159, with permission.

Table 4: Findings in those with subclinical infection at birth by age 9 years: Adverse sequelae with subclinical infection at birth

Sequelae	%
Chorioretinal lesions	86
Unilateral blindness	81
Bilateral blindness	70
Recurrent chorioretinitis	60
Severe, permanent neurologic sequelae	33
Mentally retarded	14
Sequentially lower IQ scores	86

Only 11% of children were without any of these findings. IQ: intelligence quotient.
Source: Adapted from Wilson CB, Remington JS, Stagno S, Reynolds DW (1980) Development of adverse sequelae in children born with subclinical congenital *Toxoplasma* infection. Pediatrics 66: 767-774, with permission.

Table 5. Outcomes with and without treatment. Please note also contains data relevant to outcomes with treatment.

A. Outcome of *in utero* treatment of congenitally infected fetuses with spiramycin or spiramycin followed by pyrimethamine and sulfadiazine

In Utero treatment	Patients n	Dates of Study	Dates of maternal infection[a]	Duration of follow-up	Isolates from placenta n (%)	Immune load of IgG		IgM prevalence n (%)	Subclinical infections n (%)
						at birth	at 6 mo		
Spiramycin	51	1972-1982	1972-1982	46.7 mo (2mo-11yr)	23/30 (77)	139	137	18/26 (69)	17/51 (33)
Spiramycin+ Pyrimethamine+ Sulfadiazine	52	1983-1989	1983-1989	76 wk (11-46wk)	16/38[b] (42)	86	70	8/46 (17)[c]	30/52 (57)

a: weeks gestation; b: p<0.01; c: p<0.001; mo: months; wk:weeks; yr: years.
Source: Adapted from Couvreur J, Thulliez, P., Daffos, F., Aufrant, C., Bompard, Y., Gesquiere, A., et al. (1993) *In utero* treatment of toxoplasmic fetopathy with the combination pyrimethamine-sulfadiazine. Fetal Diagnosis and Therapy 81: 45-50, with permission.

B. Transmission of *Toxoplasma gondii* infection to fetus, appearance of sequelae and severity of sequelae according to whether prenatal antibiotic therapy was given

		Time of infection (wk)		Transmission		Global sequelae[a]		Severe sequelae[a]	
	Mothers n	Mean	Range	n	%	n	%	n	%
Prenatal Treatment	119[a]	18.7	3-34	46	38.7	12	10	4	3.5
No Prenatal Treatment	25	29	6-38	18	72[b]	7	28[c]	5	20[d]
Total	144	20.5	3-38	64	44	19	13	9	6

a: four aborted fetuses are not included in the assessment of sequelae in the treated group; b: p>0.05, by multivariate analysis and controlled for gestational age; c: p=0.026, multivariate analysis and controlled for gestational age; d: p=0.007, by multivariate analysis and controlled for gestational age, wk: weeks.
Source: Adapted from Foulon W, Villena I, Stray-Pedersen B, Decoster A, Lappalainen M, et al. (1999) Treatment of toxoplasmosis during pregnancy: A multicenter study of impact on fetal transmission and children's sequelae at age 1 year. American Journal of Obstetrics and Gynecology 180: 410-415, with permission.

Table 6: Finding in US infants diagnosed following detections due to screening for increased total IgM in serum at birth: Data in 10 newborns with Congenital *Toxoplasma* infection by the presence of IgM *Toxoplasma* antibodies

Finding	No. of Infants
Maternal Illness ("flu")	2
Diagnosis suspected (neonate)	1
Gestational prematurity*	5
Intrauterine growth retardation^	2
Hepatosplenomegaly	1
Jaundice	1
Thrombocytopenia	1
Anemia	1
Chorioretinitis	2
Abnormal head size	0
Hydrocephalus	1
Microcephaly	0
Abnormal cerebrospinal fluid	8^{++}
Abnormalities on neurologic examination	1
Serum IgM elevated	9
Serum IgM *Toxoplasma* antibody	10

<37 weeks gestation; ^Lower 10th percentile (Grunewald); ++ Only eight were examined.
Source: Adapted from Alford Jr C, Stagno S, Reynolds D (1974) Congenital toxoplasmosis: clinical, laboratory, and therapeutic considerations, with special reference to subclinical disease. Bull N Y Acad Med 50: 160-181, with permission.

Table 7: New England Screening Program: Newborn manifestations at birth.

Site of Abnormality	Incidence<br>no. affected/no. examined (%)
Central nervous system	14/48 (29)*
Increased spinal fluid protein (≥ 100 mg/dl)^	8/32 (25)
Intracranial calcifications on CT scan	9/46 (20)
Enlarged lateral ventricles on CT scan or ultrasonogram	1/47 (2)
Retina	9/48 (19)
Active Chorioretinitis	2/48 (4)
Retinal scars without active inflammation	7/48 (15)
Either site	19/48 (40)[++]

*Three infants had increased protein levels and intracranial calcifications, one had intracranial calcifications and enlarged lateral ventricles, and one underwent cerebrospinal fluid evaluation but not intracranial imaging. Infants with more than 1000 red cells per cubic millimeter because of trauma due to lumbar puncture were excluded from this category

^Protein levels ranged from 100-569 mg per deciliter in cerebrospinal fluid samples obtained at three to six weeks of age, when the normal mean (±SD) concentration is 58±25mg per deciliter

++Four infants had both central version system and retinal abnormalities

Source: Adapted from Guerina NG, Hsu H, Meissner HC, Maguire JH, Lynfield R, et al. (1994) Neonatal serologic screening and early treatment for congenital toxoplasma gondii infection. New England Journal of Medicine 330: 1858-1863, with permission.

Table 8: Contrasting outcomes with fetal diagnosis and treatment

A. Prospective study of infants born to women who acquired *Toxoplasma* infection during pregnancy

Finding	Examined<br>N	Positive<br>n (%)
Prematurity	210	
birth weight <2500g		8 (3.8)
birth weight 2500-3000g		5 (7.1)
Dysmaturity (intrauterine growth retardation)		13 (6.2)
Postmaturity	108	9 (8.3)
Icterus	201	20 (10)
Hepatosplenomegaly	210	9 (4.2)
Thrombocytopenic purpura	210	3 (1.4)
Abnormal blood count (anemia, eosinophilia)	102	9 (4.4)
Microcephaly	210	11 (5.2)
Hydrocephalus	210	8 (3.8)
Hypotonia	210	2 (5.7)
Convulsions	210	8 (3.8)
Psychomotor retardation	210	11 (5.2)
Intracranial calcifications on radiography	210	24 (11.4)
Abnormal ultrasound examination	49	5 (10)
Abnormal computed tomography scan of brain	13	11 (84)
Abnormal electroencephalographic result	191	16 (8.3)
Abnormal cerebrospinal fluid	163	56 (34.2)
Micropthalmia	210	6 (2.8)
Strabismus	210	11 (5.2)
Chorioretinitis	210	
Unilateral		34 (16.1)
Bilateral		12 (5.7)

Signs and symptoms in 210 infants with proven congenital infection (1449-1960). N:300 (chorioretinisis: 76%; neurologic disease: 51%; abnormal cranial volume: 21%; calcifications: 32%).

Source: Adapted from Couvreur J, Desmonts G, Aron-Rosa D (1984) Le prognostic oculare de la toxoplasmose congenitale: role du traitement. Ann Pediatr 31, with permission.

B. Findings at birth in 55 live infants born of 52 pregnancies with prenatal diagnosis of congenital toxoplasmosis.

	n[a]	%
Subclinical infection	44/54	81
Multiple intracranial calcifications	5/54	9
Single intracranial calcification	2/54	4
Chorioretinitis scar	3/54	6
Abnormal lumbar puncture	1/54	2
Evidence o f infection on inoculation of placenta	23/46	50
Positive cord blood IgM antibody	8/53	15

A: numerator: number of abnormalities present at birth; denominator: total number of infants examined for abnormalities.
Source: Adapted from Hohlfeld P, Daffos F, Thulliez P, Aufrant C, Couvreur J, et al. (1989) Fetal toxoplasmosis: Outcome of pregnancy and infant follow-up after in utero treatment. Journal of Pediatrics 115: 765-769, with permission.

Table 9: Manifestations in infants with generalized or neurologic infection referred, in US (1959)
A: Signs, symptoms and sequelae in congenital toxoplasmosis patients

Signs and Symptoms	Frequency of occurrence in infants with (%)	
	Neurologic disease[a] n=108	Generalized disease[b] n=44
Chorioretinitis	94	66
Abnormal spinal fluid	55	84
Anemia	51	77
Convulsions	50	18
Intracranial calcifications	50	4
Jaundice	29	80
Hydrocephalus	28	0
Fever	25	77
Splenomegaly	21	90
Lympahdenopathy	17	68
Hepatomegaly	17	77
Vomiting	16	48
Microcephaly	13	0
Diarrhea	6	25
Cataracts	5	0
Eosinophilia	4	18
Abnormal bleeding	3	18
Hypthermia	2	20
Glaucoma	2	0
Optic atrophy	2	0
Microphthalmia	2	0
Rash	1	25
Pneumonitis	0	41

Presenting manifestations (a) and sequelae of congenital toxoplasmosis at ≥4 years of age (b) when generalized or neurologic manifestations were present at birth and the child was not treated.
Source: Adapted from Eichenwald HF (1960) A study of congenital toxoplasmosis, with particular emphasis on clinical manifestations, sequelae and therapy. In: Siim JC, editor. Human toxoplasmosis. Munksgaard, Copenhagen, pp. 41-49, with permission.

B. Sequelae of congenital toxoplasmosis among 105 persons followed four years or more

Condition	Neurologic<br>n=70<br>n (%)	Generalized<br>n=31<br>n (%)	Subclinical<br>n=4<br>n (%)
Mental retardation	69 (98)	25 (81)	2 (50)
Convulsions	58 (83)	24 (77)	2 (50)
Spasticity and palsies	53 (76)	18 (58)	0
Severely impaired vision	48 (69)	13 (42)	0
Hydrocephalus or microcephaly	31 (44)	2 (6)	0
Deafness	12 (17)	3 (10)	0
Normal	6 (9)	5 (16)	2 (50)

Source: Adapted from Eichenwald HF (1960) A study of congenital toxoplasmosis, with particular emphasis on clinical manifestations, sequelae and therapy. In: Siim JC, editor. Human toxoplasmosis. Munksgaard, Copenhagen, pp. 41-49, with permission.

Table 10: Transmission with treatment with Spiramycin and Pyrimethamine and Sulfadiazine treatment: Incidence of Congenital *Toxoplasma gondii* Infection by Gestational Age at Time of Maternal Infection*

Weeks of Gestation	Infected Fetuses/Total No. Fetuses	Incidence (%)
0-2	0/100	0
3-6	6/384	1.6
7-10	9/503	1.8
11-14	37/511	7.2
15-18	49/392	13
19-22	44/237	19
23-26	30/116	26
27-30	7/32	22
31-34	4/6	67
Unknown	8/351	
Total	194/2632	7.4

*Maternal Infection was treated with spiramycin in a dose of 9 million IU (3 g) daily.
Source: Adapted from Hohlfeld P, Daffos, F., Costa, J-M., Thulliez, P., Forestier, F., Vidaud, M. (1994) Prenatal diagnosis of congenital toxoplasmosis with a polymerase-chain-reaction test on amniotic fluid. N Engl J Med 695-699, with permission.

Table 11: Transmission with treatment with Spiramycin and Pyrimethamine and Sulfadiazine: Fetal *Toxoplasma* Infection as a Function of Duration of Pregnancy*

Time of Maternal Infection	No. of Women	% Infected
Periconception	182	1.2
6-16 wk	503	4.5
17-20 wk	116	17.3
21-35 wk	88	28.9
Close to term	41	75

*Women were treated during gestation as soon as feasible after diagnosis of the acute acquired infection was established or strongly suspected. If prenatal diagnosis was made in the fetus, treatment was with pyrimethamine-sulfadiazine; otherwise it was spiramycin.
Source: Adapted from Forestier F (1991) Fetal disease, prenatal diagnosis and practical measures. Presse Med: 1448-1454, with permission.

Table 12: Contrast between Congenital Toxoplasmosis in France in 2007 with treatment and earlier studies for those without treatment

France 2007 (RX), Villena		Desmonts/Couvreur (no RX)		Foulon (No RX)	
Asymptomatic	206/234=88%	Subclinical	64/94=68%	Other not specified	13/25=52%
Symptoms	21/234=8.9%	Mild	14/94=15%	Global Sequelae	7/25=28%
Severe Symptoms	7/234=3%	Severe	7/94=7%	Severe Sequelae	5/25=20%
Death/Still Born	11/272=4%	Death/Stillborn	9/94=10%	Death/stillborn	0/25=0%

Sources: Villena I, Ancelle T, Delmas C, Garcia P, Brezin AP, et al. (2010) Congenital toxoplasmosis in France in 2007: first results from a national surveillance system. Euro Surveill 15: pii-19600; Desmonts/Couvreur adapted from Desmonts G, Couvreur J. Congenital toxoplasmosis: a prospective study of the offspring of 542 women who acquired toxoplasmosis during pregnancy: pathophysiology of congenital disease. In Thalhammer O, Baumgarten K, Pollak A (eds). Perinatal Medicine, Sixty European Congress, Vienna. Stuttgart, Georg Thiem, 1979, pp 5-60, with permission; Foulon W, Villena I, Stray-Pedersen B, Decoster A, Lappalainen M, et al. (1999) Treatment of toxoplasmosis during pregnancy: A multicenter study of impact on fetal transmission and children's sequelae at age 1 year. American Journal of Obstetrics and Gynecology 180: 410-415.

Figure 1: Eye disease in congenitally infected persons detected by newborn screening (asymptomatic)

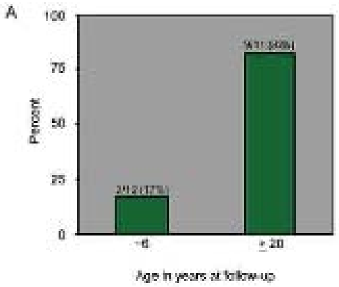

Koppe study presents visual outcome for 12 children who were asymptomatic birth, untreated, or treated less than once a month and evaluated when they were six or 20 years old. Percentage of children with retinal disease. Adverse outcomes in untreated congenital toxoplasmosis or when congenital toxoplasmosis was treated for only one month.
Sources: Koppe JG, Kloosterman GJ (1982) Congenital toxoplasmosis: long-term follow-up. Padiatr Padol 17: 171-179; Koppe JG, Loewer-Sieger DH, de Roever-Bonnet H (1986) Results of 20-year follow-up of congenital toxoplasmosis. Lancet 327: 254-256; McLeod R, Kieffer F, Sautter M, Hosten T, Pelloux H (2009) Why prevent, diagnose and treat congenital toxoplasmosis? . Memorias do Instituto Oswaldo Cruz 104: 320-344.

Figure 2: Percentage of eye disease with prompt (<u><</u> 8weeks) prenatal treatment

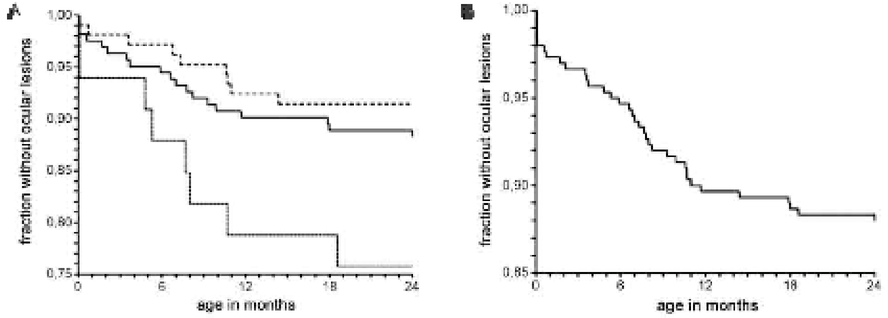

[illegible]

Source: Kieffer F, et al. (2008) Risk factors for retinochoroiditis during the first 2 years of life in infants with treated congenital toxoplasmosis. Pediatr Infect Dis J: 27-32, with permission.

Figure 3: Outcomes of NCCCTS

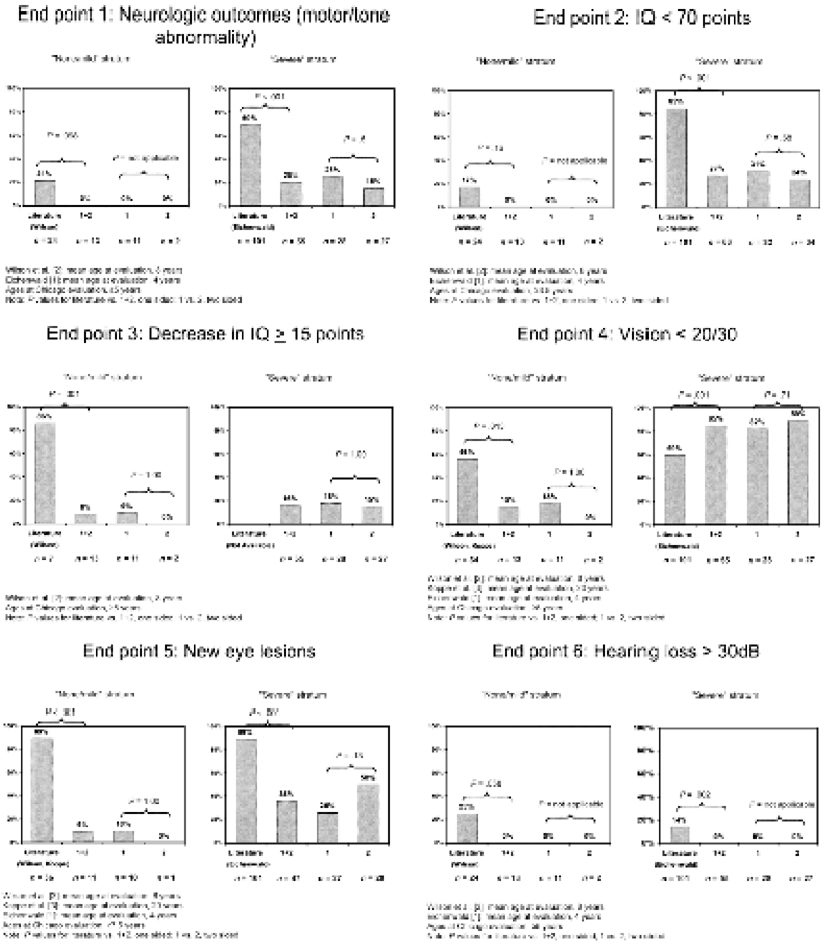

Figure 4. Frequency of outcomes for each end point for patients in our study, compared with the frequency in the literature [1–3]. There is no visible trend for superiority or statistically significant superiority for treatment arm 1 or treatment arm 2 at this time. Results may differ in the future, because the majority of the children enrolled are entering adolescence and early adulthood, a critical time when outcomes may vary. It is also important that outcomes of offspring of the treated children be established in the following years of this study. 1, Treatment arm 1; 2, treatment arm 2; IQ, intelligence quotient.

Source: McLeod R, Boyer K, Karrison T, Kasza K, Swisher C, et al. (2006) Outcome of treatment for congenital toxoplasmosis, 1981-2004: The national collaborative Chicago-based, congenital toxoplasmosis study. Clinical Infectious Diseases 42: 1383-1394, with permission.

Figure 4: Comparison of NCCCTS cohort with Eichenwald cohort in terms of severity of disease at birth

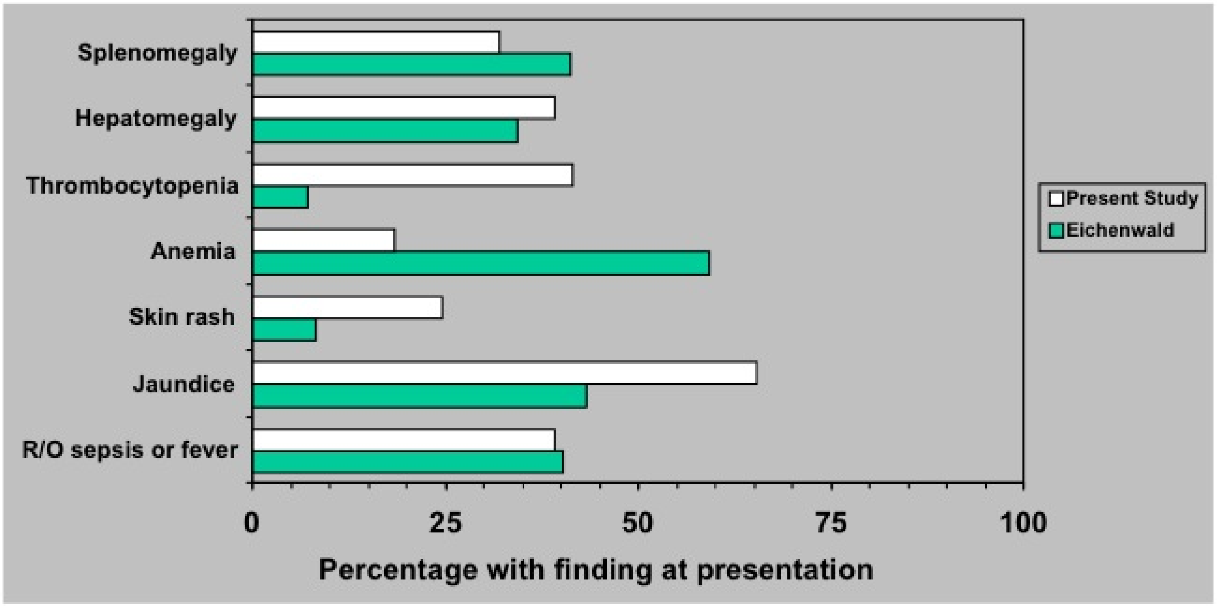

Source: McLeod R, Boyer K, Karrison T, Kasza K, Swisher C, et al. (2006) Outcome of treatment for congenital toxoplasmosis, 1981-2004: The national collaborative Chicago-based, congenital toxoplasmosis study. Clinical Infectious Diseases 42: 1383-1394, with permission.

Figure 5: Comparison of NCCCTS cohort with Eichenwald cohort in terms of severity of disease at birth

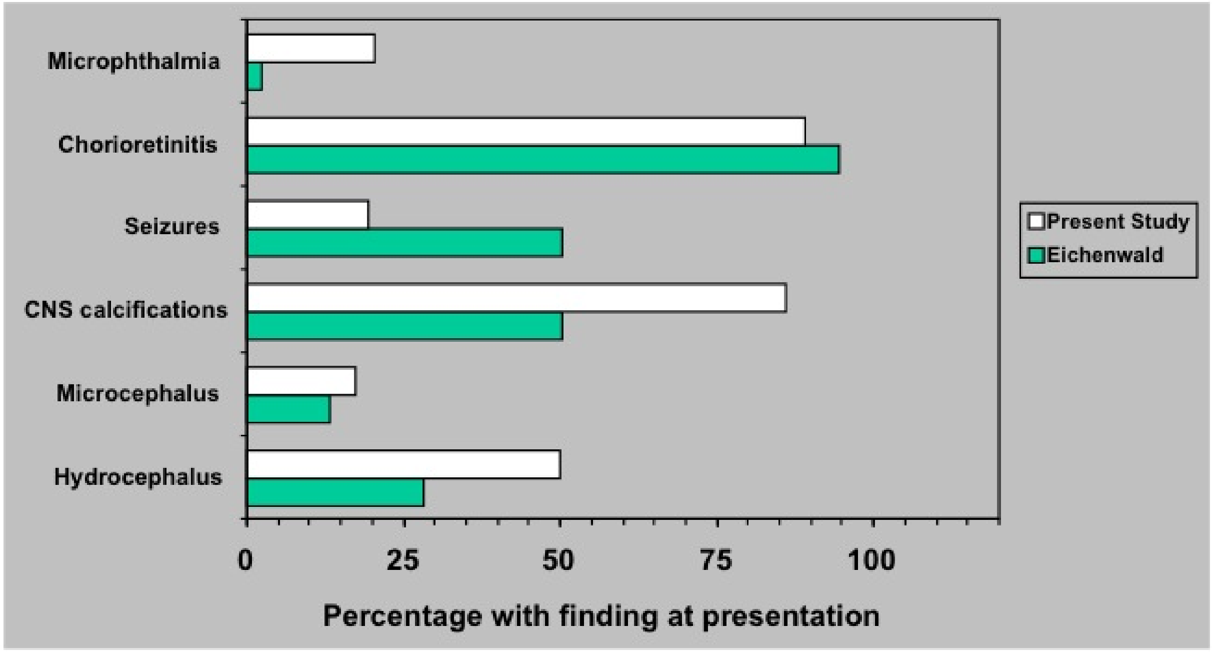

Source: McLeod R, Boyer K, Karrison T, Kasza K, Swisher C, et al. (2006) Outcome of treatment for congenital toxoplasmosis, 1981-2004: The national collaborative Chicago-based, congenital toxoplasmosis study. Clinical Infectious Diseases 42: 1383-1394, with permission.
